# The effect of postnatal steroids on lung ultrasound scores and extubation readiness in very low birth weight infants

**DOI:** 10.1038/s41372-025-02525-5

**Published:** 2026-01-05

**Authors:** Madhavi Singhal, Kate Feinstein, Michael D. Schreiber, Jeremy D. Marks, Sudhir Sriram

**Affiliations:** 1 Section of Neonatology, Department of Pediatrics, University of Chicago, Chicago, IL USA; 2 Section of Pediatric Radiology, Department of Radiology, University of Chicago, Chicago, IL USA; 3 Present Address: Department of Pediatrics, Rush University Children’s Hospital, Chicago, IL USA

**Keywords:** Respiration, Respiratory tract diseases

## Abstract

**Objective:**

We assessed the utility of lung ultrasound scores (LUSs) to predict extubation readiness in VLBW infants and determined the effect of postnatal steroids on LUSs in babies who were chronically ventilated for > 30 days.

**Study design:**

We measured infants’ LUS scores before planned extubations and determined the success of the subsequent extubation attempts.

**Results:**

Overall, LUSs were lower in successfully extubated compared with unsuccessfully extubated infants in the entire population. Similar differences were seen in LUSs between successfully and unsuccessfully extubated chronically ventilated infants. In chronically ventilated infants, LUSs did not differ between infants who did and did not receive dexamethasone. However, dexamethasone-treated infants who extubated successfully had lower scores compared to those who did not.

**Conclusions:**

While LUS scores do not predict the need for dexamethasone treatment to promote successful extubation, they do predict subsequent extubation success, irrespective of both dexamethasone treatment and duration of ventilation.

## Background

Although non-invasive ventilation is increasingly used, a significant number of very low birth weight (VLBW; birth weight <1500 grams) infants still require invasive mechanical ventilation [1, 2]. Prolonged mechanical ventilation is associated with increased mortality and morbidities, including bronchopulmonary dysplasia and neurodevelopmental delays [3, 4]. Thus, extubating mechanically ventilated infants as soon as they are ready is an important goal of care in neonatology. However, predicting extubation readiness in VLBW infants is difficult, with rates of reintubation following extubation (extubation failure) reported to be as high as 30–50% [5, 6].

Current predictors of successful extubation are unreliable, and the decision to extubate relies primarily on clinical judgment [7]. Extubation readiness tests, such as spontaneous breath tests, have added little value in assessing extubation readiness in premature infants [8], and a recent meta-analysis concluded that there is a lack of evidence to support their use [9]. Use of pneumotachography to assess respiratory load and respiratory muscle strength [10] and automated machine learning have produced promising results, but are presently difficult to incorporate into clinical use [5, 11].

More recently, point-of-care lung ultrasound (LUS) in the neonatal intensive care unit has been validated as a straightforward and quick evaluation of lung aeration and oxygenation in neonates [12, 13]. Lung ultrasound quantifies the air/fluid ratio in the lung on a region-by-region basis, and has been validated against multiple measures of lung aeration [14]. The thinness of the newborn chest wall makes identification of pleural lines, the ultrasound finding on which all evaluations are based, relatively easy [13, 15, 16]. Lung ultrasound has already been shown to be useful in predicting the need for intubation and surfactant administration in preterm infants [12, 17, 18].

In adults, LUS scores, along with the diaphragm thickening index, have proven to be good predictors of weaning success from mechanical ventilation [19]. LUS scores have also been employed to predict extubation readiness in preterm infants who were ventilated for less than 2 weeks [20–22]. However, many premature infants are difficult to wean from mechanical ventilation, so that their ventilator courses can last weeks. Such chronically ventilated babies are commonly provided with corticosteroids to either treat their chronic lung disease or promote extubation. It is not known whether LUS has a role in predicting extubation readiness in corticosteroid-treated, chronically ventilated preterm infants.

The aims of our study were to assess the utility of a neonatal-adapted LUS score to predict extubation readiness in VLBW infants and to determine the effect of postnatal steroids on LUS scores in chronically ventilated preterm infants.

## Methods

### Study setting

This was a single-center, observational, prospective study done at The Margaret M. and George A. Stephen neonatal intensive care unit (NICU) at the University of Chicago Comer Children”s Hospital. Recruitment occurred from November 2019 through May 2021 with written parental informed consent and approval by the institutional review board (IRB) at the University of Chicago. Of note, enrollment and data collection were paused for 6 months from March 2020 through September 2020 due to the SARS-CoV-2 pandemic.

### Study population

All VLBW infants who required mechanical ventilation and were admitted to the NICU were eligible for this study. Those with major congenital anomalies, including congenital heart disease, malformations of the thoracic cavity and upper airway, congenital defects of the abdominal wall, and major chromosomal abnormalities were excluded. Infants extubated within 24  hours of intubation were also excluded.

### Study design

VLBW infants intubated for respiratory distress syndrome and acute respiratory failure were enrolled. Patients were followed prospectively on a daily basis, and clinical data were collected. On the day that extubation was planned by the clinical team, the investigators were informed, and a lung ultrasound was performed within three to six hours of the extubation attempt and scored. If the patient was subsequently reintubated within seven days of the extubation attempt, the extubation was recorded as a failure. Infants were grouped according to whether the extubation was successful or not, and the LUS scores between the groups were compared.

### Ventilator management and extubation attempts

All clinical decisions, including ventilator management and decisions to extubate or re-intubate were made by the clinical team caring for the babies. The clinical team was unaware of the LUS findings.

### Lung Ultrasound examination

LUS was performed using a high-resolution L25 XP linear transducer, 6-13  MHz (Fujifilm Sonosite X Porte Ultrasound System). In each lung, aeration in each of the upper anterior, lower anterior, and lateral regions was evaluated and scored according to standardized criteria [12]. Accordingly, the total number of areas for both the right and left lungs was six. Each area was scored on a 0-3 point score system [12] and summed to obtain a neonatal adapted LUS score. The maximal summed score was thus 18. A lung that was well aerated in all zones received a score of zero, with progressively poorly aerated lung regions receiving progressively higher scores.

We used the following formula for calculating aeration of the lungs from the LUS scores:

$$	{{\rm{Aeration}}}\; {{\rm{of}}}\; {{\rm{lungs}}}\\ 	 = \frac{{{\rm{Total}}}\; {{\rm{Lung}}}\; {{\rm{Ultrasound}}}\; {{\rm{Score}}}-{{\rm{Observed}}}\; {{\rm{Lung}}}\; {{\rm{Ultrasound}}}\; {{\rm{Score}}} \times 100}{{{\rm{Total}}}\; {{\rm{Lung}}}\; {{\rm{Ultrasound}}}\; {{\rm{Score}}}}$$

### Clinical data collection

At the time of the planned extubation, baseline infant characteristics were collected, including birth weight, gestational age, gender, race, Apgar scores, and maternal characteristics, including antenatal steroids and mode of delivery. At the time of extubation, the following were recorded: medications (e.g., steroids, surfactant), duration of ventilation, weight, age, corrected gestational age, and post-extubation respiratory support. Lastly, if extubation failure did occur, the time to reintubation and the reason for reintubation were recorded.

### Data analysis

Lung ultrasound scores in the group of infants who were successfully extubated were compared with those of infants with extubation failure. Secondary analyses were done to evaluate the correlation of lung ultrasound scores with extubations in babies who did and did not receive postnatal steroids and with extubations occurring before and after 30 days of age. We also compared lung ultrasound scores among successful and unsuccessful extubations in those infants ventilated for > 30 days who received postnatal steroids. For our study, we defined infants who were ventilated > 30 days as chronically ventilated, and infants ventilated <30 days as non-chronically ventilated infants.

### Sample size estimation

Sample size was estimated based upon the area under the curve (AUC) in the receiver operating characteristic (ROC) curves. Based upon existing data in adults [19], the AUC of the ROC curve predicting successful extubation is 0.76. The sample size was calculated for a beta of 0.80, an alpha of 0.05, and the ratio of failed to successful extubation of 1 to 3. Accordingly, the total sample size required was calculated to be 46 patients.

### Statistical analysis

The normality of the data was tested by the Shapiro-Wilk test. Data are expressed in means and standard deviations, if normally distributed, and if not normally distributed, expressed in medians and interquartile ranges (IQR). We used the t-test to compare the means and the Wilcoxon rank-sum/Mann-Whitney U test to compare the medians, and the chi-square test to compare proportions. We also used logistic regression models to assess the association of successful extubation with lung ultrasound scores, controlling for potential confounders. Lastly, ROC curves with AUC were generated to predict extubation readiness. Statistical significance was set at *p*-value of <0.05. We used MedCalc to calculate sample size and STATA 17^th^ Ed for our statistical analysis.

## Results

**Fig. 1:**
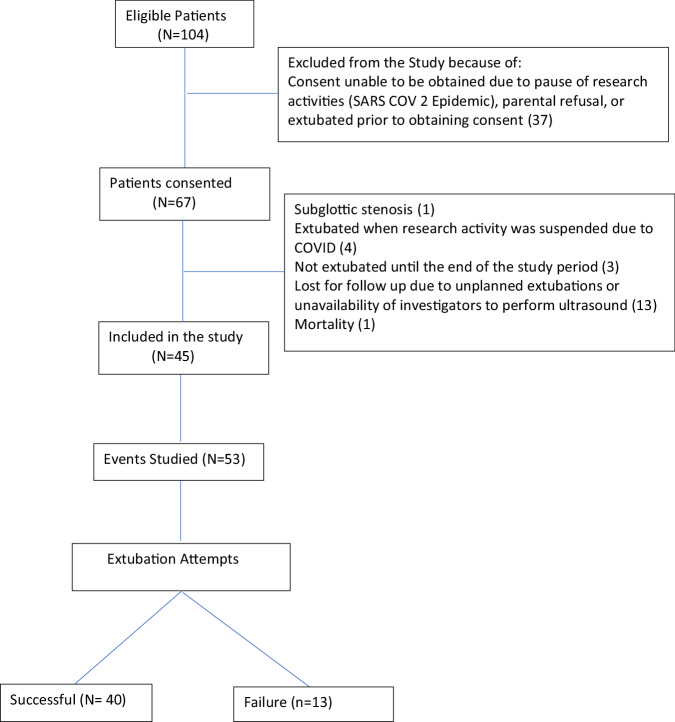
Flow chart.

During the study period, 166 VLBW infants were admitted to the NICU, of which 104 were eligible for the study (Fig. 1). Thirty-seven infants were excluded from the study because of parental refusal of consent, unavailability of investigators to perform LUS, suspension of the study due to the SARS-COV2 pandemic, or because they were extubated within 24  hours of intubation. Accordingly, 67 infants were enrolled. Of these, additional infants were excluded because they died prior to extubation (*n* = 1), had sub-glottic stenosis (*n* = 1), were extubated when research activity was suspended due to the SARS COV2 pandemic (*n* = 4), were not extubated by study end (*n* = 3), or either successfully extubated on an unplanned basis or were transferred to another hospital prior to extubation (*n* = 13). Our sample study population included 45 patients who had a total of 53 extubation events.

The mean gestational age of the population was 27 weeks ±2 (SD) and the mean birth weight 859  g ± 289 (SD). A total of 40 of 45 mothers received at least one does of antenatal steroids, and 30 of these 45 mothers (67%) received two doses. Other clinical characteristics of the infants studied appear in Table 1.

**Table 1:** Demographic Characteristics of Very Low Birth Weight (VLBW) Infants.

Characteristics	(*N* = 45)
Gestational Age, weeks, mean (SD)	27 ± 2
Birth Weight g, mean (SD)	Mean: 859 grams ± 289
Antenatal Steroids *N*, (%)	None: 5 (11%)
	Incomplete (1 dose): 10 (22%)
	Complete (≥2 doses): 30 (67%)
Vaginal/Cesarean Section *N* (%)	C-section: 35 (78%)
Race, *N* (%)	
African American	35 (78%)
Hispanic	4 (9%)
Caucasian	4 (9%)
Other	2 (4%)
Sex, N (%)	Females: 30 (67%)
One-minute APGAR score <7, *N* (%)	44 (97%)
Five-minute APGAR score <7, *N* (%)	28 (62%)
Surfactant N, (%)	
None	None: 3 (7%)
1 dose	1 Dose: 5 (11%)
2 doses	2 Doses: 20 (44%)
3 doses	3 Doses: 17 (38%)

Extubation was successful in 40 of 53 attempts (75%) and unsuccessful in 13 of 53 attempts (25%). Babies in whom extubation was unsuccessful had a lower mean gestational age (*successful*: 27 weeks ± 2  SD; *unsuccessful*: 26 weeks ± 1.5  SD, *P* = 0.01, Table 2). Notably, the mean post-conceptional age at extubation attempt in successfully extubated infants (30 week ± 2 weeks) was not significantly different from that of infants who failed extubation trials (31 ± 3 weeks). The median time until reintubation following attempts that proved to be unsuccessful was two days (1-4 days, interquartile range (IQR)). Neither birth weight nor the weight at the time of the extubation attempt differed between the groups (Table 2). Maternal factors, including the number of antenatal steroid doses and the mode of delivery, and infant factors, including doses of surfactant received, one- and five-minute Apgar scores and the age at extubation attempt did not differ between successful and failed extubation groups (Table 2).

**Table 2:** Successful vs Unsuccessful Extubation Events in VLBW Infants.

	Successful	Unsuccessful	*P* Value
Number of Events	40 (75%)	13 (25%)	
Gestational Age at Birth (weeks)	27 ± 2	26 ± 1.5	0.01*
Birth Weight (Grams)	900 ± 300	729 ± 110	0.09
Corrected Gestational Age at Extubation (Weeks)	30 ± 2	31 ± 3	0.95
Weight at Extubation (Grams)	1198 ± 447	1168 ± 328	0.88
Antenatal Steroids			
Yes	34	6	0.86
No	12	1	
Mode of Delivery			
Vaginal	10	1	0.18
C-section	30	12	
1 min Apg <7	38	13	0.56
5 min Apg <7	25	9	0.66
Surfactant Doses			
0	3		0.26
1	6		
2	19	6	
3	12	7	

Mean ± SD; * t test.

### Lung ultrasound score prior to extubation distinguishes between extubation success and failure

In the study population, the median LUS score on the day of extubation attempt was 6 (IQR: 3–11; median lung aeration 55%; IQR 83–39%). A total of 23 of 45 (51.1%) infants were successfully extubated. In infants in whom extubation failure occurred, the median lung ultrasound score was markedly and significantly higher (12; 9–12 IQR) compared with the median LUS score of infants who were successfully extubated (5; 2–8 IQR, *P* < 0.0001). Using the 75th centile of the lung ultrasound score of all infants who were successfully extubated (lung ultrasound score=8, lung aeration 56%) as the cutoff, the sensitivity of LUS scores for extubation success was 85% (95% CI: 67–95%) and specificity was 77% (95% CI 46–95%). Of note, no infant with LUS < 7 (61% aeration) failed extubation.

**Fig. 2:**
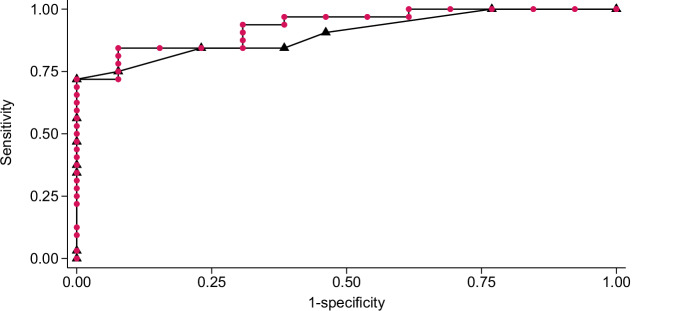
Unadjusted and Adjusted ROC curves for Lung Ultrasound Scores and Extubation Readiness in VLBW Infants (▲) Unadjusted ROC Curve: AUC 0.90 (●) Adjusted ROC Curve: AUC 0.93. Adjusted for gestational age, postnatal steroids, and ventilatory days.

We next used logistic regression to assess the association of LUS scores with successful extubation controlling for gestational age at birth, days ventilated and the use of postnatal steroids. For these analyses, we used the infants with LUS scores ≤ 6 (all of whom were successfully extubated) as the comparison group. For each unit increase in LUS score >6, we found 45% decreased odds of extubation success (adjusted OR 0.55; 0.40–0.75, *P* < 0.001). We generated receiver operating characteristic curves to estimate the area under the curve (AUC) of LUS scores to predict successful extubation (AUC 0.93, 95% CI 0.85–1.00; Fig. 2).

### Lung ultrasound scores and the duration of ventilation

Of the 23 infants who were successfully extubated on the first attempt, the median duration of ventilation was four days (IQR: 2–16 days). Of these 23 infants, 20 (87%) were extubated without corticosteroids and their median length of ventilation was three days (IQR: 2–5 days). In contrast, the 22 infants with extubation failure had a median duration of ventilation of 38 days (IQR: 6–59 days). Accordingly, we stratified infants into two categories: infants who were ventilated for fewer than 30 days (non-chronically ventilated, *N* = 29) and those who were ventilated for 30 or more days (chronically ventilated, *N* = 16, Table 3). In non-chronically ventilated infants, the median LUS score was also significantly lower in infants who were successfully extubated (3; IQR 2–6) than in those who had an extubation failure (10; IQR 8-12, *p* = 0.001).

**Table 3:** Extubations at ≤ 30 vs > 30 Postnatal Days in VLBW Infants.

	≤ 30 Postnatal Days (*N* = 29)	>30 Postnatal Days (*N* = 16)	*P* Value
Successful Extubation	23 (80%)	9 (57%)	0.10
Unsuccessful Extubation	6 (20%)	7 (43%)	
Gestational Age at Birth	28 ± 2	25 ± 2	0.001*
Birth Weight	977 ± 265	643 ± 190	0.001*
Corrected Gestational Age at Extubation	28 ± 2	33 ± 3	0.001*
Postnatal Day at Extubation	4 days (2–6)	55 days (45–65)	0.001^†^
Weight at Extubation	1005 ± 286	1456 ± 353	0.001*

Mean ± SD; Median (IQR); * t test; † Wilcoxon rank-sum/Mann-Whitney U test.

Infants who were chronically ventilated had lower gestational ages and birthweights, compared with non-chronically ventilated infants (*p* = 0.001). In contrast, their mean corrected gestational age and weight at extubation were higher compared with non-chronically ventilated infants (*p* = 0.001, Table 3). Chronically ventilated infants had higher LUS scores at extubation attempt (median 10, IQR 7–12) than did infants ventilated for fewer than 30 days (4; IQR 2–8, *P* < 0.005). In these chronically ventilated infants, the median lung ultrasound score was lower in infants who were successfully extubated (7, IQR 6–11) than in those who were not (median 12; QR 9–13, *p* = 0.02). However, infants successfully extubated after chronic ventilation had a significantly higher median LUS score than did successfully extubated infants who were not chronically ventilated (chronically ventilated: 7, IQR 6–11 vs not chronically ventilated 3; IQR 2–6, *p* = 0.002). Thus, although successful extubation is associated with significantly lower lung ultrasound scores regardless of length of ventilation, chronically ventilated infants demonstrated decreased lung aeration at successful extubation.

### Lung ultrasound scores after dexamethasone treatment to promote extubation

No babies ventilated for <30 days received postnatal steroids. Although steroids were provided on an individualized basis, in general, steroids were considered for premature infants who were not extubated by 30 days of age, who were on ventilator support from which extubation would fail, and who weighed more than 1.0  kg. Accordingly, we asked how dexamethasone treatment of infants ventilated for more than 30 days affected extubation and LUS scores. Of the 16 chronically ventilated infants, 12 (75%) received postnatal steroids. Of these, two infants (17%) received peri-extubation dexamethasone for presumed airway edema [23], and 10 infants (83%) received postnatal dexamethasone using the DART protocol [24].

Median lung ultrasound scores in chronically ventilated infants who received postnatal dexamethasone (10, IQR 8–12) were not different from scores of chronically ventilated infants who did not receive postnatal dexamethasone (9, IQR 6-12). Notably, only five of 12 babies who received postnatal dexamethasone (42%) were successfully extubated. Although LUS scores in chronically ventilated infants did not distinguish between dexamethasone-treated and untreated infants, lung ultrasound scores did distinguish dexamethasone-treated infants who were successfully extubated from dexamethasone-treated infants who were not: The median LUS score of the five infants who were successfully extubated following dexamethasone (7, IQR 6–8) was lower than the median score of the seven dexamethasone-treated infants (58%), who were not successfully extubated (12, IQR 9–13, *p* < 0.02).

Of the dexamethasone-treated infants, no infant with LUS < = 7 failed extubation. In dexamethasone-treated infants, for each unit increase in LUS score >8, we found 54% decreased odds of extubation success (adjusted OR 0.46; 0.21–0.99, *P* < 0.05). Using the 75^th^ centile of the lung ultrasound score of the successfully extubated babies (lung ultrasound score = 8, lung aeration 56%) as the cutoff, the sensitivity of LUS scores for extubation success was 80% (95% CI: 28%, 99%), and the specificity was 85% (95% CI 42–97%). We generated ROC curves to estimate the AUC of LUS scores to predict successful extubation (AUC 0.93, 95% CI 0.79–1.00).

## Discussion

Our study shows that the neonatal-adapted lung ultrasound score, performed on the same day as a planned extubation, is an excellent predictor of extubation success in mechanically ventilated VLBW infants. In addition, dexamethasone treatment of the subpopulation of chronically ventilated babies is not associated with lower lung ultrasound scores compared with those of untreated chronically ventilated infants.

Our findings are consistent with those of several prospective cohort studies [20–22] demonstrating the utility of lung ultrasound scores in predicting extubation success in premature infants ventilated for five to seven days. Thus, El Amrousy et al. [20] studied 80 infants using the same methodology [12]. However, in contrast with the present study, they found that a LUS of 4 (78% aeration) constituted the cutoff for successful extubation. Our finding of a higher cutoff value of 8 (56% aeration) is likely due to the larger percentage of extremely preterm infants in our population compared with that study’s population which included infants < 35 weeks gestation. In contrast, Soliman et al. [21] obtained LUS scores on the third and seventh days of life and found markedly higher median lung ultrasound scores at extubation (11.5/18) equivalent to lung aeration of 36%, compared with the median lung aeration value of 56% that we observed in our study. These differences may reflect earlier extubation to non-invasive ventilation than in use in our clinical setting. In our clinical setting, infants were routinely extubated to CPAP only. In contrast, infants in the Soliman et al. study, infants were extubated to either CPAP or non-invasive ventilation based on their work of breathing. More recently, Mohsen et al. [22] obtained pre-extubation LUS scores in 44 infants with gestational age <28 weeks. In contrast to the present study, these authors employed a different scoring system, such that they observed extubation success at a lung ultrasound score of 15/24, equivalent to lung aeration of 38%. This degree of lung aeration, allowing successful extubation was much poorer than the 56% lung aeration cutoff we observed. Unlike infants in the present study, infants in the Mohsen et al. study were extubated to nasal positive pressure ventilation. Non-invasive, positive pressure ventilation is more likely to reduce the loss of lung functional residual capacity over time after extubation [25, 26] compared with CPAP, and reduce the risk of extubation failure. Finally, differences in lung ultrasound scores, which are a proxy for lung aeration in predicting extubation success, may also be partially due to differences between the study populations’ respiratory disease severity, which is influenced by genetic factors [27].

We found that dexamethasone treatment of chronically ventilated babies is not associated with lower lung ultrasound scores compared with those of chronically ventilated infants who successfully extubate without dexamethasone treatment. That is, LUS scores in infants who extubated successfully without dexamethasone were not, contrary to our anticipations, significantly lower than those of infants who required dexamethasone for successful extubation. This finding suggests that the degree of lung aeration, however achieved, is the key determinant of extubation success. However, it is possible that, had LUS scores been obtained in dexamethasone-treated infants prior to dexamethasone treatment, those scores would have been significantly higher compared with infants who extubated without dexamethasone treatment. Finally, because the number of infants who extubated without dexamethasone treatment was low, it is possible that the absence of a significant difference in LUS scores between groups is due to the limited power of the analysis.

Overall, LUS scores of chronically ventilated infants did predict subsequent extubation success, irrespective of both dexamethasone treatment and duration of ventilation. Our findings are congruent with those of a recent Italian study of the effect of dexamethasone/budesonide treatment on LUS scores [28]. In this study, of the 12 VLBW infants undergoing invasive mechanical ventilation, dexamethasone (9 of 12) or budesonide inhalation (3 of 12) resulted in marked decreases in LUS scores (from a median of 10 to 5) and increased lung aeration (from a median of 44% to 72%). Notably, the cumulative dose of dexamethasone employed in that study (2.625  mg/kg) is almost threefold higher than the cumulative dexamethasone dosage provided in the DART protocol we and most centers employ in the US.

The findings of our single-center study are consistent with those of previous single-center, prospective studies, and support the use of LUS scores to define extubation readiness in VLBW infants. The increasing availability of point-of-care ultrasound will provide real-time, non-invasive determination of an infant”s readiness for extubation, reducing the complications associated with failed extubation.

## Conclusions

Lung ultrasound scoring is an excellent predictor of extubation readiness in VLBW infants, even in chronically ventilated infants. However, lung ultrasound scores are higher in chronically ventilated (>30 days) babies, demonstrating poorer lung aeration. While LUS scores do not predict the need for dexamethasone treatment to promote successful extubation, they do predict subsequent extubation success, irrespective of both dexamethasone treatment and duration of ventilation.

## Summary

### Article summary

- Lung ultrasound scores are shown to be excellent predictors of extubation readiness in VLBW infants. Lung ultrasound scores are higher in chronically ventilated infants (> 30 days), compared with infants ventilated < 30 days. LUS scores do not predict the need for dexamethasone treatment to promote successful extubation in chronically ventilated infants.

### What is known

- Lung ultrasound scores can predict extubation readiness in preterm infants who have been ventilated for <2 weeks duration.

### What this adds

- Lung ultrasound scores predict extubation readiness in babies ventilated >30 days. In chronically ventilated infants, postnatal steroid treatment does not differentiate LUSs from those of infants not treated with steroids. However, lung ultrasound scores predict extubation success in infants treated with postnatal steroids.

## Data Availability

Non-identified datasets analyzed during the current study are available from the corresponding author on reasonable request.

## References

[1] Morley CJ, Davis PG, Doyle LW, Brion LP, Hascoet J-M, Carlin JB. Nasal CPAP or intubation at birth for very preterm infants. N Engl J Med. 2008;358:700–8. 18272893 10.1056/NEJMoa072788

[2] SUPPORT Study Group of the Eunice Kennedy Shriver NICHD Neonatal Research Network. Early CPAP versus surfactant in extremely preterm infants. N Engl J Med. 2010;362:1970–9. 20472939 10.1056/NEJMoa0911783 PMC3071534

[3] Walsh MC, Morris BH, Wrage LA, Vohr BR, Poole WK, Tyson JE, et al. Extremely low birthweight neonates with protracted ventilation: mortality and 18-month neurodevelopmental outcomes. J Pediatr. 2005;146:798–804. 15973322 10.1016/j.jpeds.2005.01.047

[4] Jensen EA, DeMauro SB, Kornhauser M, Aghai ZH, Greenspan JS, Dysart KC. Effects of multiple ventilation courses and duration of mechanical ventilation on respiratory outcomes in extremely low-birth-weight infants. JAMA Pediatrics. 2015;169:1011–7. 26414549 10.1001/jamapediatrics.2015.2401 PMC6445387

[5] Shalish W, Sant”Anna GM, Natarajan G, Chawla S. When and how to extubate premature infants from mechanical ventilation. Curr Pediatr Rep. 2014;2:18–25.

[6] Chawla S, Natarajan G, Shankaran S, Carpenter B, Brion LP, Kezler M, et al. Markers of successful extubation in extremely preterm infants, and morbidity after failed extubation. J Pediatr. 2017;189:113–9.e112. 28600154 10.1016/j.jpeds.2017.04.050 PMC5657557

[7] Al-Mandari H, Shalish W, Dempsey E, Keszler M, Davis P. Sant’‘Anna G. International survey on periextubation practices in extremely preterm infants. Arch Dis Child-Fetal Neonatal Ed. 2015;100:F428–31. 26063193 10.1136/archdischild-2015-308549

[8] Shalish W, Kanbar L, Kovacs L, Chawla S, Kezler M, Rao S, et al. Assessment of extubation readiness using spontaneous breathing trials in extremely preterm neonates. JAMA Pediat. 2020;174:178–85. 10.1001/jamapediatrics.2019.4868 PMC6990705 31860014

[9] Shalish W, Latremouille S, Papenburg J, Sant’‘Anna GM. Predictors of extubation readiness in preterm infants: a systematic review and meta-analysis. Arch Dis Child Fetal Neonatal Ed. 2019;104:F89–97. 29519808 10.1136/archdischild-2017-313878

[10] Dimitriou G, Greenough A, Endo A, Cherian S, Rafferty G. Prediction of extubation failure in preterm infants. Arch Dis Child Fetal Neonatal Ed. 2002;86:F32–5. 11815545 10.1136/fn.86.1.F32 PMC1721344

[11] BrasherM VirdovA, Raffay TM, Bada HS, Cunningham MD, Bumgardner C, et al. Predicting Extubation Readiness in Preterm Infants Utilizing Machine Learning: A Diagnostic Utility Study. J Pediatr. 2024;271:114043. 38561049 10.1016/j.jpeds.2024.114043 PMC12055302

[12] Brat R, Yousef N, Klifa R, Reynaud S, Aguilera SS, De Luca D. Lung ultrasonography score to evaluate oxygenation and surfactant need in neonates treated with continuous positive airway pressure. JAMA Pediatr. 2015;169:e151797. 26237465 10.1001/jamapediatrics.2015.1797

[13] Lichtenstein DA, Mauriat P. Lung ultrasound in the critically ill neonate. Curr Pediatr Rev. 2012;8:217–23. 23255876 10.2174/157339612802139389 PMC3522086

[14] Sartorius V, Brunet S, De Luca D. Characteristics of scores used for quantitative lung ultrasound in neonates: a systematic review. Eur Respir Rev. 2025;34:240232. 40240059 10.1183/16000617.0232-2024 PMC12000906

[15] Kurepa D, Zaghloul N, Watkins L, Liu J. Neonatal lung ultrasound exam guidelines. J Perinatol. 2018;38:11–22. 29144490 10.1038/jp.2017.140

[16] Raimondi F, Yousef N, Migliaro F, Capasso L, De Luca D. Point-of-care lung ultrasound in neonatology: classification into descriptive and functional applications. Pediatr Res. 2021;90:524–31. 30127522 10.1038/s41390-018-0114-9 PMC7094915

[17] De Martino L, Yousef N, Ben-Ammar R, Raimondi F, Shankar-Aguilera S, De Luca D Lung ultrasound score predicts surfactant need in extremely preterm neonates. Pediatrics. 2018;142. 10.1542/peds.2018-0463 30108142

[18] Raimondi F, Migliaro F, Sodano A, Ferrara T, Lama S, Vallone G, et al. Use of neonatal chest ultrasound to predict noninvasive ventilation failure. Pediatrics. 2014;134:e1089–94. 25180278 10.1542/peds.2013-3924

[19] Tenza-Lozano E, Llamas-Alvarez A, Jaimez-Navarro E, Fernández-Sánchez J. Lung and diaphragm ultrasound as predictors of success in weaning from mechanical ventilation. Crit Ultrasound J. 2018;10:1–9. 29911284 10.1186/s13089-018-0094-3 PMC6004341

[20] El Amrousy D, Elgendy M, Eltomey M, Elmashad AE. Value of lung ultrasonography to predict weaning success in ventilated neonates. Pediatr Pulmonol. 2020;55:2452–6. 32609928 10.1002/ppul.24934

[21] Soliman RM, Elsayed Y, Said RN, Abdulbaqi AM, Hashem RH, Aly H. Prediction of extubation readiness using lung ultrasound in preterm infants. Pediatr Pulmonol. 2021;56:2073–80. 33819393 10.1002/ppul.25383

[22] Mohsen N, Nasef N, Ghanem M, Yeung T, Deekonda V, Ma C, et al. Accuracy of lung and diaphragm ultrasound in predicting successful extubation in extremely preterm infants: A prospective observational study. Pediatr Pulmonol. 2023;58:530–9. 36324211 10.1002/ppul.26223

[23] Davis PG, Henderson-Smart DJ. Intravenous dexamethasone for extubation of newborn infants. Cochrane Libr. 2001;4:CD000308. 10.1002/14651858.CD000308 11687075

[24] Doyle LW, Davis PG, Morley CJ, McPhee A, Carlin JB. DART StudyInvestigators Low-dose dexamethasone facilitates extubation among chronically ventilator-dependent infants: a multicenter, international, randomized, controlled trial. Pediatrics. 2006;117:75–83. 16396863 10.1542/peds.2004-2843

[25] Ramaswamy VV, More K, Roehr CC, Bandiya P, Nagina S. Efficacy of noninvasive respiratory support modes for primary respiratory support in preterm neonates with respiratory distress syndrome: systematic review and network meta-analysis. Pediatr Pulmonol. 2020;55:2940–63. 32762014 10.1002/ppul.25011

[26] Lemyre B, Deguise MO, Benson P, Kriplani H, Ekhaguere OA, Davis PG. Early nasal intermittent positive pressure ventilation (NIPPV) versus early nasal continuous positive airway pressure (NCPAP) for preterm infants. Cochrane Database Syst Rev. 2023;19:CD005384. 10.1002/14651858.CD005384.pub3 PMC10355255 37466143

[27] Shen CL, Zhang Q, Hudson JM, Cole FS, Wambach JA. Genetic factors contribute to risk for neonatal respiratory distress syndrome among moderately preterm, late preterm, and term infants. J Pediatr. 2016;172:69–74.e62. 26935785 10.1016/j.jpeds.2016.01.031 PMC4876036

[28] Rigotti C, Zannin E, Chiaraluce S, Ventura ML. Lung function response to postnatal corticosteroids for the prevention and treatment of bronchopulmonary dysplasia. Pediatr Res. 2025;97:1605–11. 39242937 10.1038/s41390-024-03535-3

